# Phase I/II trial of BMS-986,205 and nivolumab as first line therapy in hepatocellular carcinoma

**DOI:** 10.1007/s10637-023-01416-w

**Published:** 2023-12-01

**Authors:** Jasmine C. Huynh, May Cho, Arta Monjazeb, Ebaa Al-Obeidi, Amisha Singh, Kit Tam, Frances Lara, Anthony Martinez, Leslie Garcia, Edward J. Kim

**Affiliations:** 1 Division of Hematology and Oncology, Davis Comprehensive Cancer Center, University of California, Sacramento, CA 95817 USA; 2 Division of Hematology and Oncology, Irvine Comprehensive Cancer Center, University of California, Orange, CA 92868 USA; 3 Department of Radiation Oncology, Davis Comprehensive Cancer Center, University of California, Sacramento, CA 95817 USA; 4 Department of Internal Medicine, University of California, Davis Medical Center, Sacramento, CA 95817 USA; 5 Office of Clinical Research, Davis Comprehensive Cancer Center, University of California, Sacramento, CA 95817 USA

**Keywords:** Hepatocellular carcinoma, Immunotherapy, Nivolumab, Indoleamine 2,3-dioxygenase

## Abstract

**Background:**

Indoleamine-2,3-dioxygenase (IDO) helps orchestrate immune suppression and checkpoint inhibitor resistance in hepatocellular carcinoma (HCC). BMS-986,205 is a novel oral drug that potently and selectively inhibits IDO. This Phase I/II study evaluated the safety and tolerability of BMS-986,205 in combination with nivolumab as first-line therapy in advanced HCC.

**Methods:**

Adults with untreated, unresectable/metastatic HCC received BMS-986,205 at two dose levels (50–100 mg orally daily) in combination with fixed dose nivolumab (240mg/m^2^ IV on Day 1 of each 14-day cycle). The primary objective was to determine the safety and tolerability of this combination; secondary objectives were to obtain preliminary efficacy.

**Results:**

Eight patients received a total of 91 treatment cycles in the dose escalation phase. All patients were Child Pugh A and 6 patients had underlying viral hepatitis. In the 6 evaluable patients, no dose-limiting toxicities (DLTs) were observed. The most common treatment-related adverse events (TRAEs) were aspartate transaminase (AST) and alanine transaminase (ALT) elevation (3 patients) and diarrhea, maculopapular rash and increased alkaline phosphatase (2 patients each). Grade 3 events were diarrhea and AST elevation (1 patient), and hyperglycemia and pancreatitis requiring treatment discontinuation (1 patient). No grade 4–5 events occurred. Partial response was observed in 1 patient (12.5%) and stable disease in 3 patients (37.5%), yielding a disease control rate of 50%. Median PFS was 8.5 weeks; median OS was not reached.

**Conclusion:**

Combination BMS-986,205 and nivolumab showed a manageable safety profile with durable benefit as first-line therapy in a meaningful subset of advanced HCC patients.

## Introduction

Hepatocellular carcinoma (HCC) is a leading cause of cancer-related mortality with more than 500,000 new cases per year worldwide [1–3]. Unfortunately, most HCC patients present with advanced disease precluding them from curative therapies such as surgical resection or locoregional treatment [4]. Sorafenib was approved by the FDA in 2007, and for a decade it remained the first and only line of treatment for unresectable HCC. Beginning in 2017, other tyrosine kinase inhibitors (TKIs) including regorafenib, cabozantinib and lenvatinib were approved (as first or second line therapy) [5–7], as well as immune checkpoint inhibitors given alone or in combination with vascular endothelial growth factor inhibitors (VEGFi) or TKIs [8]. Despite this, prognosis for patients with advanced HCC remains poor with a median overall survival (mOS) of 12–19 months. New treatment strategies are urgently needed that go beyond targeting VEGF and immune checkpoints [5, 6, 8].

Poor treatment response in HCC is partly due to HCC arising in a background of liver cirrhosis and chronic inflammation. The underlying liver dysfunction both complicates treatment toxicity and serves as an independent, competing factor in patient prognosis. Unique to the setting of cirrhosis are persistent inflammatory signals that result in pathological changes within the liver stroma, such as the influx of regulatory T-cells (Tregs) and myeloid derived suppressor cells (MDSCs), dampened cytotoxic T-cell activity and upregulation of programmed death-ligand 1 (PD-L1) expression [9–12]. Binding of PD-L1 to programmed death-1 (PD-1) receptors on T cells leads to T cell anergy and exhaustion, increased Treg differentiation, and metabolic alterations that promote tumor growth [13–16]. This abundance of immunosuppressive factors in the HCC tumor microenvironment presents an opportunity for immunotherapeutic approaches [16].

Nivolumab is a fully humanized monoclonal antibody against PD-1 currently approved as second-line therapy for the treatment of advanced HCC [14]. Response rates to nivolumab monotherapy in HCC are under 20%, partially due to the development of resistance [17]. Indoleamine 2,3-dioxygenase (IDO) is an intracellular enzyme involved in the rate limiting step of the catabolism of tryptophan to kynurenine, which is expressed by multiple human malignancies, including HCC [18, 19]. IDO plays a central role in orchestrating immune suppression within the tumor microenvironment and can induce immune tolerance to malignancies [20]. IDO expression can also be paradoxically upregulated after inflammatory signals, presumably as a mechanism to limit inflammation and maintain immune homeostasis. A series of studies have demonstrated the role of IDO in the immune escape of multiple types of tumors, specifically correlating with reduced intratumoral T cell infiltration, disease progression, and shorter survival [21]. In HCC, increased expression and activation of IDO has been associated with both the early and late phases of liver carcinogenesis. HCC tumors with IDO overexpression have also been associated with poor prognosis [22, 23]. BMS-986,205 is a novel oral drug that potently and selective inhibits human IDO, and thus can reduce immune tolerance and resistance to immune checkpoint blockade [20, 21, 24]. Therefore, combining IDO inhibition and PD-L1 inhibition is a promising therapeutic strategy.

Preliminary clinical reports have shown synergy and relative safety for combining IDO inhibitors and immune checkpoint blockade in other malignancies. The combination of IDO inhibitor epacadostat and pembrolizumab produced an objective response rate (ORR) of 56% and a disease control rate (DCR) of 71% in untreated melanoma patients in phase I/II of the ECHO-202/KEYNOTE-037 trial. The most common adverse events were rash (46%) and fatigue (43%) [25]. A phase I/II study of BMS-986,205 with nivolumab demonstrated efficacy in advanced bladder and cervical cancer patients with ORR of 32% and 14%, respectively [26]. The combination was well tolerated with only 4 patients (1.4%) discontinuing the study due to drug-related toxicities, and most frequent adverse effects included fatigue, nausea, and decreased appetite.

Based on the activity of immune checkpoint blockade in HCC, the mechanistic rationale for combining IDO inhibitors and immunotherapy, and the tolerability of this combination in other malignancies, we conducted a Phase I/II trial evaluating the safety and efficacy of BMS-986,205 in combination with nivolumab in unresectable HCC.

## Methods

This clinical trial (NCT 03695250) was conducted following all applicable regulatory requirements and was approved by the UC Davis Institutional Review Board (IRB).

### Patient selection

Eligible patients were age ≥ 18 years with unresectable or metastatic, histologically or imaging-confirmed hepatocellular carcinoma (HCC) which was not amenable to curative treatment approach. Patients must have had measurable disease by RECIST v.1.1 criteria, and ≥ 1 liver lesions accessible for core biopsy that was either untreated with prior liver-directed therapy or progressed following liver-directed therapy. Patients had to have Child-Pugh score A at enrollment, Eastern Cooperative Oncology Group (ECOG) performance status of 0–1, and able to swallow intact pills. Patients with active hepatitis B virus (HBV) were permitted if antiviral therapy for hepatitis had been administered for ≥ 8 weeks and viral load was < 100 IU/mL prior to the first dose of trial treatment; patients with untreated hepatitis C virus (HCV) were permitted. Other eligibility criteria included: absolute neutrophil count (ANC) of ≥ 1000 cell/mm^3^, platelet count of ≥ 50,000/mm^3^, hemoglobin ≥ 8 g/dL, total bilirubin and creatinine ≤ 2x the institutional upper limit of normal (ULN), and aspartate aminotransferase (AST), alanine aminotransferase (ALT), and alkaline phosphatase < 5x the institutional ULN. Patients had to undergo mandatory pre-treatment biopsy in cases where insufficient archival tumor specimen was available as well as on-treatment biopsy.

Key exclusion criteria included receipt of any current or prior systemic cancer-related therapy, immunodeficiency history, active autoimmune disease or diseases/disorders requiring a systemic steroid equivalent of prednisone ≥ 10 mg/day or immunosuppressive therapies given within 7 days before the first dose of the study, any active bacterial, fungal or viral infections (excluding HBV and HCV), and any known history of pneumonitis. Patients could not receive liver directed therapy within 4 weeks of the first dose of study drug. Patients were also ineligible if there was clinically significant ascites, hepatic encephalopathy, esophageal/gastric varices with bleeding within 3 months of study enrollment, receipt of live attenuated vaccines within 30 days of first study treatment, additional malignancies, G6PD deficiency or other congenital/autoimmune hemolytic disorders, history or presence of cytochrome b5 reductase deficiency, QTc interval > 480ms and any treatment with botanical preparations within two weeks prior to randomization.

All subjects provided written consent and the study was compliant with Good Clinical Practices guidelines and the Declaration of Helsinki.

### Study design and objectives

This was an open-label Phase I/II clinical trial. Phase I was conducted as a 3 + 3 dose escalation study and Phase II was planned as a Simon two-stage design to allow for early trial stoppage for futility. The primary objectives of this study were to determine the safety and tolerability of BMS-986,205 in combination with nivolumab in unresectable/metastatic HCC in the first line setting using CTCAE V5.0 criteria, and to determine efficacy of this combination using RECIST (version 1.1) criteria. Secondary objectives included obtaining preliminary data on disease control rate (DCR), duration of response (DOR), ORR using immune RECIST (iRECIST) criteria, progression free survival (PFS), and OS, and to further evaluate the safety of this combination.

For the Phase I dose-escalation portion, the primary endpoint was to determine the safety profile of BMS-986,205 in two dose levels (50 mg and 100 mg) with fixed-dose nivolumab 240mg/m^2^ IV every 14 days. Patients were treated following a standard 3 + 3 design, with dose level 1 (DL1) defined as BMS-986,205 50 mg daily and dose level 2 (DL2) as BMS-986,205 100 mg daily, in order to determine the maximum tolerated dose (MTD). MTD was defined as the dose at which ≤ 1 out of 6 patients develops a DLT at BMS-986,205 DL1 or DL2. DLT was defined as any drug-related Grade 2 uveitis or eye pain requiring systemic therapy or unresponsive to topical therapy and failing to improve within 2 weeks of starting therapy, any Grade 2 drug-related pneumonitis or interstitial lung disease unresponsive to dose delay and systemic steroids within 2 weeks, any Grade 3 non-skin drug-related adverse event (excluding laboratory abnormalities, fatigue and nausea) unrelieved or controlled with appropriate care within two weeks, any Grade 4 drug-related adverse events including laboratory abnormalities (except Grade 4 leukopenia or neutropenia) lasting < 14 days, methemoglobin levels ≥ 15% and Grade ≥ 3 hemolysis (i.e., requiring transfusion or medical intervention such as steroids), and any study drug-related Grade ≥ 3 hemolysis requiring transfusion or steroids. The following drug-related hepatic function laboratory abnormalities were also considered DLTs: AST or ALT more than 10 times the upper limit of normal for more than two weeks, AST or ALT more than 15 times the upper limit of normal (irrespective of duration), and total bilirubin greater than five times the upper limit of normal (irrespective of duration). To be evaluable for DLT, the patient must have received at least 75% of the total intended dose of BMS-986,205 during the first 6 weeks of therapy. Therapy was continued until progression or 24 months, with the option to continue therapy after 24 months per treating physician discretion.

The Phase II portion was planned to be a Simon two-stage design with a total of 17 patients, inclusive of 6 patients from the Phase I portion treated at the MTD.

### Safety and efficacy assessments

Safety assessments consisted of monitoring and recording all adverse events, including serious adverse events, lab parameters and physical exam at each study visit. Toxicity was evaluated according to National Cancer Institute Common Terminology Criteria for Adverse Events (NCI CTCAE, version 5.0 criteria). All patients receiving any amount of study drug were evaluable for toxicity.

Response was assessed using computed tomography (CT) scans of chest, abdomen, and pelvis at baseline and then at the end of 8 weeks, 16 weeks, and then every 12 weeks per Response Evaluation Criteria in Solid Tumors (RECIST) guideline (version 1.1) as well as immune RECIST (iRECIST) criteria. DOR, PFS and OS were evaluated using Kaplan-Meier plots.

Adverse events were summarized according to organ system, laboratory category, and dose level in frequency tables graded according to CTCAE v5.0. Information regarding each subject’s course including completion of therapy, dose delays, premature discontinuation, and major protocol violations were tabulated and summarized.

### Statistical considerations

This was a phase I/II study with a planned Simon optimal two-stage design for efficacy evaluation in Phase II. In Phase I, patients were treated following a standard 3 + 3 design with dose escalation in BMS-986,205 and fixed-dose nivolumab. In Phase II dose expansion, initially three additional patients were to be enrolled at the MTD for a total of 9 patients at the MTD, including the 6 patients treated at the MTD in the Phase I portion. An interim efficacy analysis was to be done of those 9 patients and if ≥ 1 of 9 initial patients treated at MTD achieved a response, 8 more patients would be accrued at the MTD. If ≥ 3 of 17 patients treated at MTD achieved a response, the treatment would be deemed worthy of further study, provided the safety profile is acceptable. The Simon optimal two-stage design provided 80% power to detect the difference between an acceptable response rate by RECIST v1.1 of 25% vs. an unacceptable rate of 5% at the 0.05 level (1sided). ORR was estimated as the proportion of participants who experienced an objective response, along with its exact 95% confidence interval. DCR was analyzed similarly. DOR, PFS, and OS were analyzed using Kaplan-Meier methods, and medians and 95% confidence intervals were computed. Safety analyses were tabulated for each patient and summarized in frequency tables.

## Results

### Clinical characteristics

From November 2018 to December 2019, a total of 8 patients were enrolled in the Phase I dose-escalation cohort at the UC Davis Comprehensive Cancer Center. Patient characteristics are shown in Table 1. The median age of patients was 69 years (range 60–76) and 6 patients were men. The majority of patients were White (5 patients) and had a baseline European Cooperative Oncology (ECOG) score of 1 (5 patients). At baseline, most patients were Barcelona Clinic Liver Cancer (BCLC) Stage A (5 patients) with median alpha fetoprotein (AFP) level ≤ 400 µg/L (6 patients), and all patients were Child Pugh (A) Three patients had a history of hepatitis C and 1 patient had a history of hepatitis (B) No patients had received prior systemic therapy, but 4 patients received prior local therapies, consisting of lobectomy, radiofrequency ablation, ethanol ablation, transarterial chemoembolization (TACE), and selective internal radiation therapy (SIRT).

**Table 1:** Baseline demographic and clinical characteristics of study patients

Baseline Characteristics	
Characteristic	All Patients (n = 8) n (%)
**Median age**, years (range)	69 (60–76)
**Sex**	
Male	6 (75%)
Female	2 (25%)
**Race**	
White	5 (63%)
Asian	2 (25%)
African American	1 (13%)
**ECOG** ^**a**^	
0	3 (38%)
1	5 (63%)
**BCLC Stage** ^**b**^	
A	5 (63%)
B	1 (13%)
C	2 (25%)
**Child Pugh Score**	
A	8 (100%)
B	0 (0%)
**Hepatitis C Status**	
Positive	5 (63%)
Negative	3 (38%)
**Hepatitis B Status**	
Positive	1 (13%)
Negative	7 (88%)
**Prior systemic treatment**	
Yes	0 (0%)
No	8 (100%)
**Prior local therapy**	
Yes	4 (50%)
No	4 (50%)
**Baseline AFP level**	
≥ 400 µg/L	2 (25%)
≤ 400 µg/L	6 (75%)

^a^ECOG: Eastern Cooperative Oncology Group; ^b^BCLC: Barcelona clinic liver cancer

### Treatment exposure

Three patients were treated at DL1 and 5 patients were treated at DL2. A total of 91 cycles of therapy (range 2–43) were administered; the longest duration of therapy was 43 cycles in patient 005 who was treated at DL2, followed by 27 cycles in patient 002 who was treated at DL1.

For BMS-986,205, four patients required a dose hold/omission due to adverse events. Patient 002, treated at DL1, required 3 dose holds in total; 2 holds were due to diarrhea that was initially concerning for immune-related colitis but then attributed to food poisoning, and 1 hold was due to immune-related hepatitis with Grade 3 AST and Grade 2 elevation. Patient 006 required a dose hold/omission due to Grade 2 diarrhea which was initially concerning for immune-related colitis but then was attributed to laxative use and self-resolved. Patient 007 required a dose delay/omission due to hospitalization for a new spinal mass and discontinued the clinical trial prior to further therapy. Patient 009 required a dose hold/omission due to Grade 2 elevation in AST and ALT. Patients 006, 007 and 009 were all treated at DL2.

#### Dose-limiting toxicities and MTD

Six of the eight patients were evaluable for DLT with 3 patients evaluated at each dose level; 2 patients were replaced for DLT assessment due to insufficient dose administered and this was unrelated to toxicity. No DLTs were observed at either dose level. MTD was not determined due to insufficient number of DLT-evaluable patients treated at DL2.

### Efficacy evaluation

**Fig. 1:**
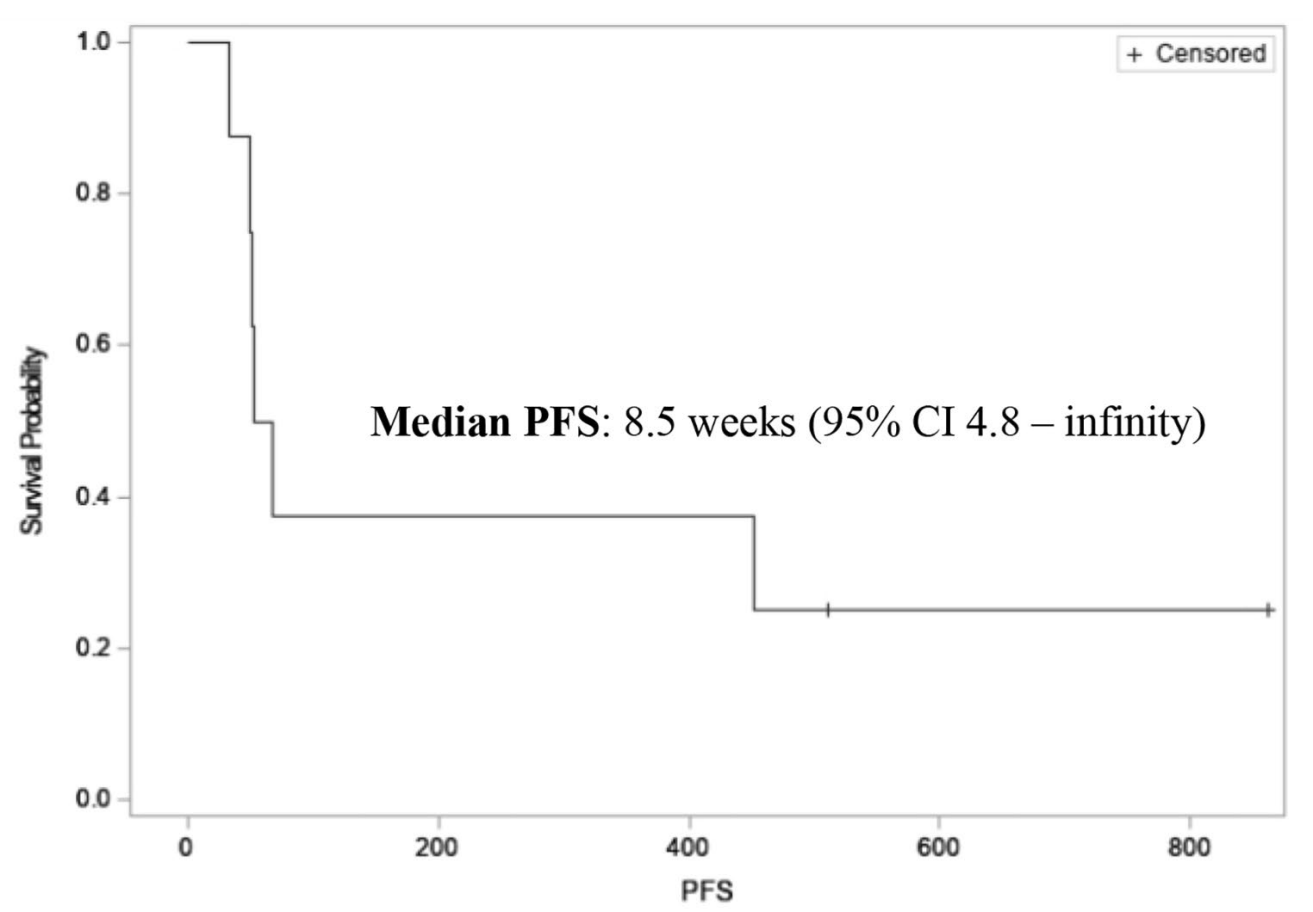
Kaplan-meier curve illustrating median progression-free survival (PFS)

**Fig. 2:**
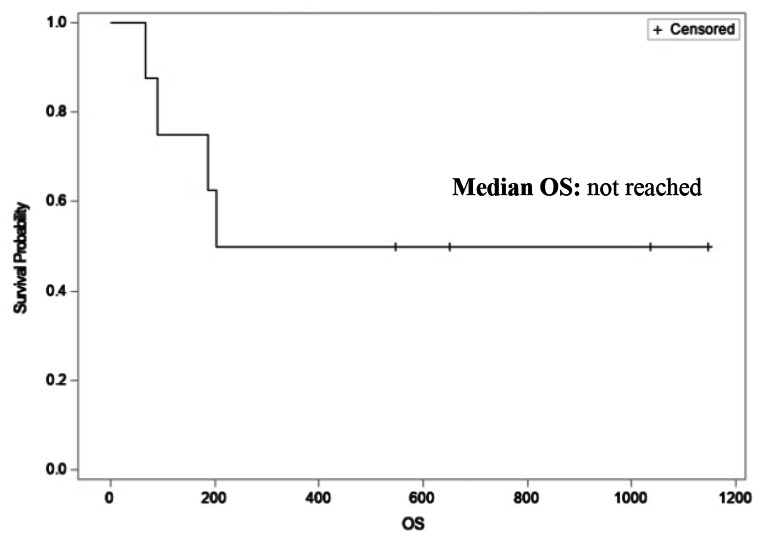
Kaplan-meier curve illustrating median overall survival (OS)

All eight patients were evaluable for efficacy. By RECIST criteria, the ORR was 12.5% with the best response of PR in 1 patient (12.5%), SD in 3 patients (37.5%) and PD in 4 patients (50%), as shown in Table 2. DCR was 50%. By iRECIST criteria, best response was iUPD in 4 patients (50%). Median PFS was 8.5 weeks (95% CI 4.8 – infinity, Fig. 1) and median OS has not been reached (Fig. 2).

**Table 2:** Efficacy endpoints and measures of best response

Best response using RECIST criteria	
Response	All patients (n = 8) n (%)
Complete Response	0 (0%)
Partial Response	1 (12.5%)
Stable Disease	3 (37.5%)
Progressive Disease	4 (50%)
Disease control rate	50%

### Safety

All 8 patients were evaluable for safety. A total of 24 treatment-related adverse events (TRAEs) occurred and TRAEs were observed in all patients (Table 3). The most common TRAEs of all grades were AST elevation and ALT elevation in 3 patients, diarrhea in 2 patients, maculopapular rash in 2 patients and increased alkaline phosphatase in 2 patients. A total of 4 Grade 3 events were observed, which included diarrhea and AST elevation in patient 002, and hyperglycemia and pancreatitis requiring treatment discontinuation in patient 009. Grade 3 events occurred in patients treated at both dose levels. There were no Grade 4 events. Four patients died due to disease progression; there were no treatment-related deaths.

**Table 3:** Reported treatment-related adverse events by Grade

Treatment-related adverse events		
**Event, N (%)**	**All patients (n = 8)**	
**Adverse Event**	**Any Grade**	**Grade ≥ 3**
Diarrhea	2 (25%)	1 (12.5%)
Oral pain	1 (12.5%)	0
Flatulence	1 (12.5%)	0
Pruritus	1 (12.5%)	0
Fatigue	1 (12.5%)	0
Dyspnea	1 (12.5%)	0
Maculopapular rash	2 (25%)	0
Alk phos increase	2 (25%)	0
AST increase^a^	3 (37.5%)	1 (12.5%)
ALT increase^b^	3 (37.5%)	0
Musculoskeletal pain	1 (12.5%)	0
Anorexia	1 (12.5%)	0
Malaise	1 (12.5%)	0
TSH elevation^c^	1 (12.5%)	0
Abdominal pain	1 (12.5%)	0
Hyperglycemia	1 (12.5%)	1 (12.5%)
Pancreatitis	1 (12.5%)	1 (12.5%)

^a^AST: aspartate aminotransferase; ^b^ALT: alanine transaminase; ^c^TSH: thyroid stimulating hormone

A total of 4 serious adverse events (SAEs) occurred in two patients. Patient 002, who was treated at DL1, had Grade 3 diarrhea and Grade 3 AST increase, which was also considered an immune related adverse event which improved with holding treatment. Patient 009, who was treated at DL2, had hyperglycemia (Grade 3) and pancreatitis (Grade 3), and this required treatment discontinuation.

## Discussion

This study evaluated the safety, tolerability and preliminary efficacy of IDO inhibitor BMS-986,205 and PD-1 inhibitor nivolumab in patients with unresectable/metastatic HCC. The combination was found to have an acceptable safety profile with manageable TRAEs; there were no DLTS, only 2 patients experienced grade 3 adverse events, and no patients experienced grade 4–5 TRAEs. The disease control rate was 50% overall with stable disease as best response in 3 patients. One patient treated at DL2 (BMS986205 100 mg daily and nivolumab 240mg/m^2^ IV on D1 of a 14-day cycle) achieved a durable partial response such that he received a total of 43 cycles of therapy on study.

The rationale for combination IDO inhibitor and PD-L1 therapy stems from preclinical data which has shown that the IDO pathway may contribute to primary and acquired resistance of PD-L1 inhibitors. Both IDO and PD-L1 are upregulated by interferon signaling, suggesting that PD-L1 positive tumors may also up-regulate IDO [27–29]. In HCC, increased expression and activation of IDO is associated with liver carcinogenesis, and persistent IDO expression within the liver microenvironment may play a critical role in declining HBV and HCV specific T-cell response 22, 23, 30, 31]. In murine HCC models, immune checkpoint inhibitors led to an increase in IDO expression by HCC tumor cells via an IFN-γ-dependent mechanism, and this promoted resistance to single-agent CTLA-4 inhibition and was overcome with IDO inhibitor 1-methyl-D-tryptophan [32]. Additionally, blocking IFN-γ in mice who received anti-CTLA-4 therapy also suppressed the induction of IDO in the tumor microenvironment, suggesting that the effector mechanism of immune checkpoint blockade may be mediated by IFN-γ [32]. Taken together, this provides the basis for clinical investigation of the combination of IDO inhibitor and PD-L1 blockade.

Clinically, combination IDO inhibitor and PD-L1 blockade has been tested in several tumor types. Early phase clinical trials of this combination demonstrated encouraging results but this trend did not persist in later-phase studies. The Phase I/II ECHO-202/KEYNOTE-037 evaluated IDO inhibitor epacadostat with pembrolizumab in patients with advanced solid tumors. Notably, the 22 patients with advanced melanoma had a remarkable response rate of 55%^25^. However, in the confirmatory randomized Phase III ECHO-301/KEYNOTE-252 trial, combination epacadostat and pembrolizumab failed to meet its primary endpoint of improved PFS compared to pembrolizumab monotherapy in patients with advanced melanoma [33]. A leading hypothesis of why the confirmatory trial failed is that the Phase III trial was conducted too soon, without supporting evidence from a randomized Phase II study – the Phase III trial was based on a signal of activity in a small group of patients in the Phase I/II study. The disappointing results of the ECHO-301/KEYNOTE-252 halted other IDO inhibitor plus immune checkpoint blockade trials in melanoma and dampened enthusiasm for IDO inhibitors. In our study, which was also based on a small number of patients, combination BMS-986,205 and nivolumab exhibited a tolerable safety profile with partial response in 1 patient and stable disease in 3 patients.

Limitations to our study include the small sample size of 8 patients. Additionally, our study was designed when sorafenib was the only FDA-approved first-line therapy, but during the conduct of the study, new agents became available (lenvatinib, atezolizumab/bevacizumab) and altered the landscape of first-line therapy for advanced HCC [6, 34]. We reacted to this shifting landscape by amending the protocol to allow patients with one prior line of therapy. However, despite this, we only enrolled one additional patient after the amendment and that patient was a previously untreated patient; thus, all 8 patients enrolled were treatment-naïve. Combination atezolizumab/bevacizumab has since become the new standard of care first-line treatment for patients as it is better tolerated and demonstrated a notable mOS benefit (HR 0.58; 95% CI: 0.42, 0.79; p = 0.0006) and mPFS benefit (HR 0.59; 95% CI: 0.47, 0.76; p < 0.0001) compared to sorafenib alone [8]. With the introduction of atezolizumab/bevacizumab as a viable and effective first-line therapy option for advanced HCC patients, accrual to our study was challenging. This highlights the difficulties of conducting clinical trials in the rapidly-changing landscape of metastatic HCC treatment.

The approval of atezolizumab/bevacizumab marked the start of the shifting landscape to multi-drug treatment regimens for metastatic HCC. Other multi-drug trials include COSMIC-312 which was a Phase III trial evaluating atezolizumab/cabozantinib versus sorafenib as first-line treatment for unresectable advanced HCC patients and it demonstrated improved PFS but not OS [35]. LEAP-002 was a Phase III trial that evaluated pembrolizumab/lenvatinib versus single-agent lenvatinib as first-line treatment for unresectable advanced HCC and did not find a statistically significant improvement in PFS or OS [36]. The lenvatinib control arm performed better than expected which was felt to have contributed to the lack of statistical significance – median OS was 21.2 months vs. 19.0 months and ORR 40.8% vs. 34.1%. Dual checkpoint blockade has also been studied in two phase III trials of patients with unresectable advanced HCC: HIMALAYA and CheckMate 9DW. The HIMALAYA trial evaluated tremelimumab/durvalumab versus sorafenib and found a statistically significant OS benefit compared to sorafenib alone (16.4 months vs. 13.8 months, HR 0.78, p = 0.0035) and is now FDA approved as a first line therapy option [37]. CheckMate 9DW is comparing first-line nivolumab/ipilimumab to standard of care TKI (sorafenib or lenvatinib), and results are not yet published [38]. Single agent checkpoint inhibitors have also been studied in the first line setting such as in CheckMate 459, a phase III trial of nivolumab vs. sorafenib which did not show an improvement in OS and RATIONALE-301, a phase III trial of tislelizumab (PD-1 inhibitor) vs. sorafenib which showed noninferiority in OS but a shorter PFS in the tislelizumab arm [39, 40].

Though our study was a small, single-arm study with limited efficacy, the findings add evidence for another combination immunotherapy strategy in the treatment landscape of unresectable HCC. As more combination therapies have emerged, questions of how best to sequence therapy and how to identify which subsets of patients will benefit most from each therapy are now a pressing challenge. Future studies focused on determining biomarkers of response to therapy will be beneficial.

In conclusion, our study demonstrated that the combination of BMS-986,205 and nivolumab is tolerable with a manageable safety profile. However, due to the challenges to accrue in the setting of rapidly-changing treatment options for HCC, we were unable to completely determine the true MTD or efficacy of this particular combination. Despite the many treatment options now available for HCC, there remains a limited number of lines of therapy as the treatments can be grouped into either VEGF-targeted therapy or immune checkpoint inhibition. This challenge will only be heightened as additional doublet combinations of TKI/checkpoint inhibitor and dual checkpoint inhibitor trials yield results that mirror or surpass atezolizumab and bevacizumab, but then further reduce actual number of lines of therapy. It is in this context that studies such as ours investigating novel targets will be needed to continue to truly expand the armamentarium against advanced HCC.

## Data Availability

The datasets generated during and/or analyzed during the current study are available from the corresponding author on reasonable request.
